# Lipidomics and Transcriptomics Differ Liposarcoma Differentiation Characteristics That Can Be Altered by Pentose Phosphate Pathway Intervention

**DOI:** 10.3390/metabo12121227

**Published:** 2022-12-07

**Authors:** Zhengqing Song, Shuaikang Wang, Lili Lu, Jingshen Xu, Qiwen Zhou, Weiqi Lu, Hanxing Tong, Yong Zhang, Wenshuai Liu, Zhiming Wang, Wei Li, Yang You, Chenlu Zhang, Xi Guo, Rongkui Luo, Yingyong Hou, Chunmeng Wang, Yuexiang Wang, Lei Sun, He Huang, Yuhong Zhou

**Affiliations:** 1 Department of Medical Oncology, Biotherapy Center, Department of General Surgery, Department of Pathology, Zhongshan Hospital, Shanghai Key Laboratory of Metabolic Remodeling and Health, Institute of Metabolism and Integrative Biology, Institute of Developmental Biology and Molecular Medicine, Fudan University, Shanghai 200032, China; 2 Department of Musculoskeletal Oncology, Fudan University Shanghai Cancer Center, Shanghai 200032, China; 3 Institute of Nutritional and Health Science, Chinese Academy of Sciences, Shanghai 200031, China; 4 Shanghai Qi Zhi Institute, Shanghai 200030, China

**Keywords:** liposarcoma, lipidomics, transcriptomics, adipogenic differentiation, pentose phosphate pathway

## Abstract

Liposarcoma (LPS) is a rare and heterogeneous malignancy of adipocytic origin. Well-differentiated liposarcoma (WDLPS) and dedifferentiated liposarcoma (DDLPS) are two of the most common subtypes, showing similar genetic characterizations but distinct biological behaviors and clinical prognosis. Compared to WDLPS, DDLPS is more aggressive and has the potential of metastasis, as the malignant adipocytic tumor’s metabolic changes may have taken place during the tumorigenesis of LPSs. Therefore, to investigate the lipid alterations between the two subtypes, high-resolution liquid chromatography tandem mass spectrometry (LC-MS/MS) based untargeted lipidomic analysis was performed onto LPS tissues from 6 WDLPS and 7 DDLPS patients. The lipidomic analysis showed the upregulated phosphatidylcholines and phosphoethanolamines in DDLPS, and the upregulated triglycerides and diglycerides in WDLPS, which might be due to the uncompleted adipocytic dedifferentiation leading to such tumorigenesis. Such a finding was also confirmed by the similarity comparison of two LPS subtypes to the transcriptome of stromal vascular fraction at different differentiation stages. Transcriptomic analysis also demonstrated that metabolic pathways including the pentose phosphate pathway (PPP) were upregulated in WDLPS compared to DDLPS. Therefore, the cell line LPS853 was treated with the PPP inhibitor 6-aminonicotinamide ex vivo and the proliferation and invasion of LPS853 was significantly promoted by PPP inhibition, suggesting the potential role of PPP in the development and differentiation of LPS. In conclusion, this study described the altered lipid profiles of WDLPS and DDLPS for the first time, revealing the different differentiation stages of the two subtypes and providing a potential metabolic target for LPS treatment.

## 1. Introduction

Liposarcoma (LPS) is a kind of rare and heterogeneous malignant adipocytic tumor [1]. Well-differentiated liposarcoma (WDLPS) and dedifferentiated liposarcoma (DDLPS) are the two most common subtypes, which usually occur within the retroperitoneum [2]. The diagnosis of WDLPS or DDLPS mainly depends on their pathological characteristics. Both WDLPS and DDLPS are malignant adipocytic tumors characterized by the amplification of *MDM2* and *CDK4*, which is usually confirmed by fluorescence in situ hybridization (FISH) [3]. Although WDLPS and DDLPS share similar genetic characterizations, they show distinct biological behaviors and clinical prognosis. To distinguish between WDLPS and DDLPS, the histological hallmark of WDLPS is the presence of adipocytes resembling normal adipose tissue, among which disperse stromal spindle cells with nuclear atypia [4]. While the term DDLPS was introduced in 1979 to define the morphologic progression from WDLPS to a non-lipogenic sarcoma [5], which is mainly composed of high-grade spindle cells with heterologous differentiation, showing few apparent adipocytic characteristics[3]. In addition, although the local recurrence rates are both high in WDLPS and DDLPS, WDLPS has no metastatic potential while DDLPS is more aggressive. DDLPS has increased risk of recurrence and metastasis [4], with poorer disease-free survival (DFS) and overall survival (OS) from resection [5]. According to the nomogram of LPS established by Dalal et al., the 5-year disease-specific survival (DSS) for WDLPS, which was classified as the low-grade, was 93% (CI, 88–95%), while that for the high-grade DDLPS was only 44% (CI, 33–54%) [6]. Similar results have also been found by our center that FNCLCC grade was related to the prognosis of retroperitoneal LPS [7]. Surgical resection is the prioritized treatment for localized WDLPS or DDLPS. For patients with advanced or unresectable tumors, anthracycline-based chemotherapy is the standard management though either WDLPS or DDLPS shows low sensitivity to chemotherapy [8]. Clinical trials of new drugs, such as target therapy and immunotherapy, have been carried out recently, but most of them only showed limited effect on DDLPS [9,10,11]. The high recurrence rate and poor prognosis urgently calls for additional therapeutic strategies.

Although it is observed in both WDLPS and DDLPS that the amplifications of Chr 12q could encode oncogenes, including *MDM2*, *CDK4* and *YEATS2* [2], two genes (*HMGA2* and *CPM*) that affect adipocyte and mesenchymal cell differentiation are frequently rearranged in DDLPS tumors [12]. Considering that WDLPS and DDLPS both have gross tumor volumes without specific supplying vessels within the tumors, it suggests that they may have unique metabolic features different from most tumors. WDLPS and DDLPS are considered to be of adipocytic origin and WDLPS consist of a large portion of adipocytes; the analysis of lipid metabolism might clarify the differences between these two LPS subtypes. In this study, we collected 6 WDLPS and 7 DDLPS tumor samples to perform lipidomic experiments using a high-resolution liquid chromatography tandem mass spectrometry (LC-MS/MS) technique. The lipidomics provide lipid metabolism differences between two LPS subtypes, which might directly relate to the adipocytic differentiation. Combining the genomic analysis, we found the perturbation of the metabolic pathway could indeed alter the LPS cell behavior.

## 2. Results

### 2.1. Lipidomics Reveals the Lipid Alterations between WDLPS and DDLPS

**Figure 1:**
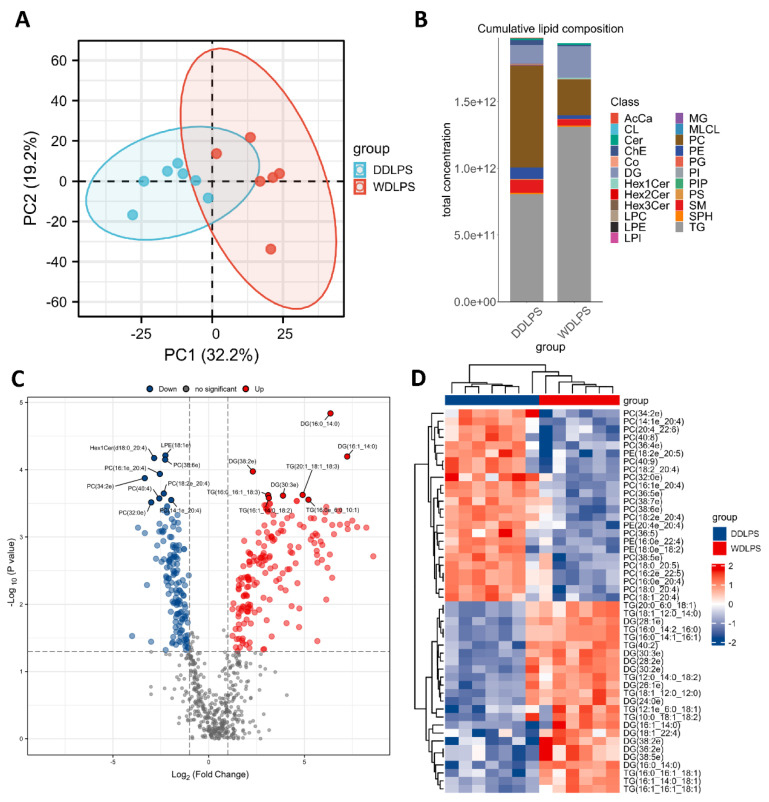
Lipid profiles of WDLPS and DDLPS tumors. (**A**) The PCA score plot of the two subtypes of tumors. Each blue dot in (A) represents a DDLPS tumor, and each red dot represents a WDLPS tumor. (**B**) The stacked histogram of cumulative lipid classes in the two subtypes of tumors. (**C**) The volcano plot showing the significantly changed lipids in WDLPS compared to DDLPS. (D) The heatmap showing the top 48 altered lipids in WDLPS and DDLPS.

A total of 13 patients received surgical treatment in Zhongshan Hospital, Fudan University and pathologically diagnosed with WDLPS or DDLPS were included in this study. The clinical information of these patients was shown in Table 1. Gross pictures and H&E staining of all the samples were shown in Figure S1. To investigate the alterations of lipid metabolism between the two subtypes of LPS, we performed lipidomics experiments on WDLPS (n = 6) and DDLPS (n = 7) tumors from respective patients using high-resolution LC-MS/MS. Principal component analysis (PCA) revealed a difference of lipidome on two groups (Figure 1A). Both WDLPS and DDLPS tumors were mainly composed of phosphocholines (PCs), triacylglycerides (TGs), diacylglycerides (DGs), cholesterol (ChE), sphingomyelin (SM) and phosphoethanolamines (PEs). Compared to DDLPS, there were much more DG and TG, as well as fewer PC, PE, SM and ChE in WDLPS tumors (Figure 1B). Such increased DG and TG implied that the WDLPS tumors contained more lipid droplets, which were more similar to the adipose tissues. We then took a close look at these altered lipids using a volcano plot. The volcano plot showed 180 upregulated and 143 downregulated lipids in WDLPS compared to DDLPS (fold change >1 or fold change <−1 and *p*-value < 0.05) (Figure 1C). The remarkably upregulated lipids in WDLPS included DG(16:0/14:0), DG(16:1/14:0), TG(16:0e/6:0/10:1), TG(20:1/18:1/18:3), DG(30:3e), TG(16:1/14:0/18:2), TG(16:0/16:1/18:3) and DG(38:2e), while lipids including PC(34:2e), PC(32:0e), Hex1Cer(d18:0/20:4), PC(40:4), PC(16:1e/20:4), PC(18:2e/20:4), PC(38:6e), LPE(18:1e) and PC(14:1e/20:4) were significantly downregulated in WDLPS. The heatmap showed the distinct lipid profiles between WDLPS and DDLPS, which also confirmed the increase of TG and DG, and the decrease of PC and PE in WDLPS (Figure 1D). The lipidomic analysis results indicated that the main difference between two types of LPS tumors might be due to their differentiation stage difference. Lipidomic was also performed on normal fat and large lipomas to explore whether it was different from that of WDLPS. As shown in the PCA plot (Figure S2A), the lipid composition of both lipoma and WDLPS were generally similar to that of normal fat. Although the Venn diagram showed that the lipidomics of lipoma was generally different from WDLPS (Figure S2B), it could be learnt from Figure S3 that TG, DG and LPE were both upregulated and PE and SM were both downregulated in WDLPS and lipoma compared to normal fat.

### 2.2. Transcriptome Reveals That WDLPS and DDLPS Correlate to Different Adipocytic Differentiation Stages

**Figure 2:**
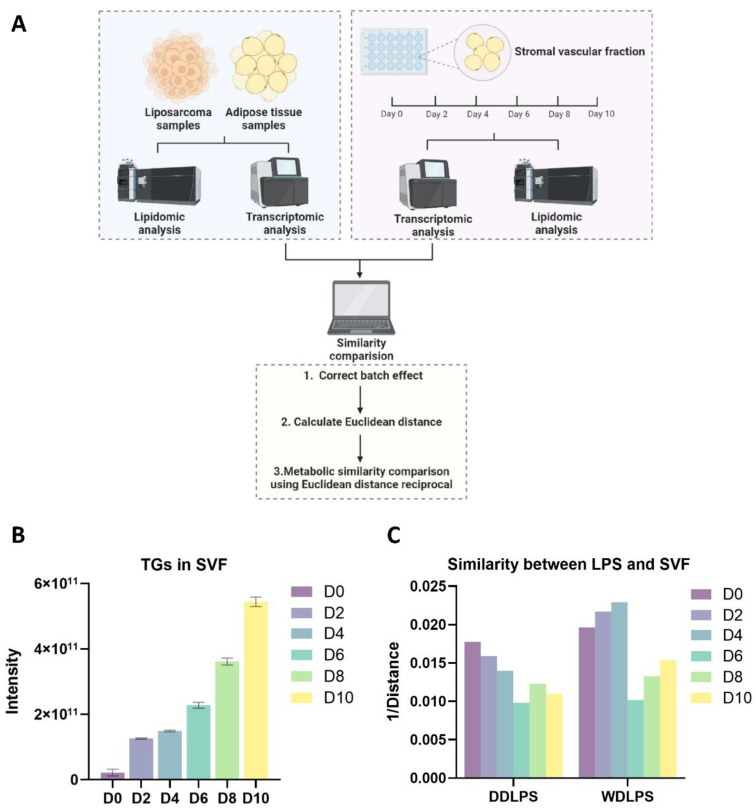
Metabolic similarity comparison between LPS and SVF. (**A**) Overview of the strategy used to compare the transcriptome of LPS tissues and SVF cells. (**B**) The accumulated TGs in SVF cells from day 0 to day 10. (**C**) The bar plot showing the similarity between LPS tumors and SVF cells on day 0, day 2, day 4, day 6, day 8 and day 10. (The figure **A** was created with BioRender.com, accessed on 10 October 2022).

To investigate whether the WDLPS and DDLPS might be at different adipocytic differentiation stages, the transcriptomic analysis would be the best tool. RNA-sequencing was also performed on these samples to further identify the adipocytic features of WDLPS and DDLPS. In addition, we set a standard adipocytic differentiation cellular model as a reference. Mouse stromal vascular fraction (SVF), a cell model of adipogenesis being widely studied [13], was established in our previous study [14]. SVF was isolated from a C57BL/6J mouse and differentiated into mature adipocytes ex vivo using an adipocyte differentiation cocktail from day 0 to day 10 (Figure 2A). SVF cells gradually accumulated lipid droplets and differentiated from the preadipocytes to the mature adipocytes [14]. The lipidomic analysis conducted on these cells showed the gradually accumulated TGs in SVF cells from day 0 to day 10 (Figure 2B), which indicated the maturation of adipocytes. Since SVF cells on each time points represented different adipocytic differentiation stages, the transcriptome of tumor tissues was compared to that of the SVF cells on day 0, day 2, day 4, day 6, day 8 and day 10 using a calculated Euclidean distance (Equation (1)). Each bar in Figure 2C represented a calculated Euclidean distance reciprocal, and the higher bar meant a higher similarity between tumor samples and the SVF cells in gene expression manner. In general, these two LPS subtypes were more similar to a relatively early stages of adipocyte as the similarity bars were higher on day 0 to 4, and lower on day 6 to 10. The WDLPS tumor transcriptome was more similar to the gene expression of SVF on day 4 but DDLPS was closer to the day 0 SVF gene expression, indicating the different adipocytic differentiation stages of DDLPS and WDLPS, as we expected.

### 2.3. Transcriptomic Analysis Reveals the Upregulation of Pentose Phosphate Pathway in WDLPS

**Figure 3:**
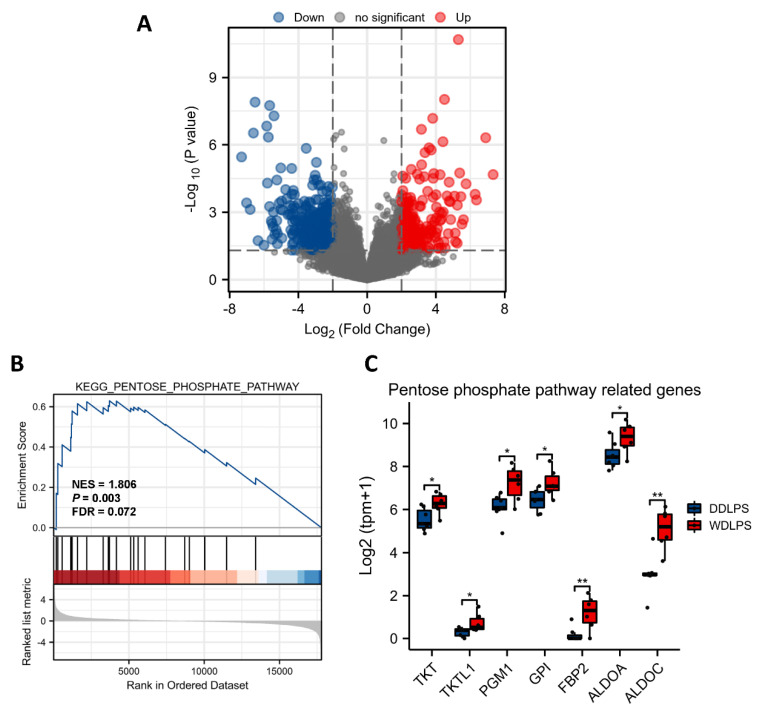
Transcriptomic features of WDLPS. (A) The volcano plot showing significantly changed genes in WDLPS compared to DDLPS. (**B**) GSEA indicated the significant upregulation of the pentose phosphate pathway in WDLPS. (**C**) Upregulated genes related to the pentose phosphate pathway in WDLPS. *, *p* < 0.05; **, *p* < 0.01.

As the transcriptome results indicated the WDLPS and DDLPS tumor differences were related to their different adipocytic differentiation stages, we then took a close look at their gene expression differences. After performing quality control and data normalization, a total of 23,951 genes were identified. The volcano plot showed the gene expression was different between WDLPS and DDLPS tumor samples (Figure 3A). By analysis of the differentially expressed genes (DEGs), 417 upregulated genes and 219 downregulated genes were observed among the two groups, based on the cut-off criteria (fold change >2 or fold change <−2 and *p*-value < 0.05). To further explore the differential metabolic pathways between WDLPS and DDLPS, gene set enrichment analysis (GSEA) using the RNA-seq data was performed. A total of 14 KEGG metabolic pathways were obtained (NES >1 or NES <−1 and *p*-value < 0.05) (Figure S4), among which the pentose phosphate pathway (PPP) was one of the most significantly upregulated one in WDLPS (Figure 3B). The expression of differential genes related to PPP, including TKT, TKTL1, PGM1, GPI, FBP2, ALDOA and ALDOC, were all upregulated in WDLPS tumor samples (Figure 3C), which were validated by Real-time Quantitative PCR (RT-qPCR) (Figure S5A). When compared WDLPS with normal fat or lipoma, there was no significant difference between the expression of these genes (Figure S5B,C). It is well-known that the PPP is highly linked to the cell proliferation and differentiation processes [15,16]. Therefore, it may be upregulated in normal fat and well differentiated adipocytic tumors. Thus, we hypothesized that the perturbation of such pentose phosphate metabolism could change the LPS tumor behavior.

### 2.4. Inhibition of Pentose Phosphate Pathway Promotes Cell Proliferation and Invasion of Liposarcoma

**Figure 4:**
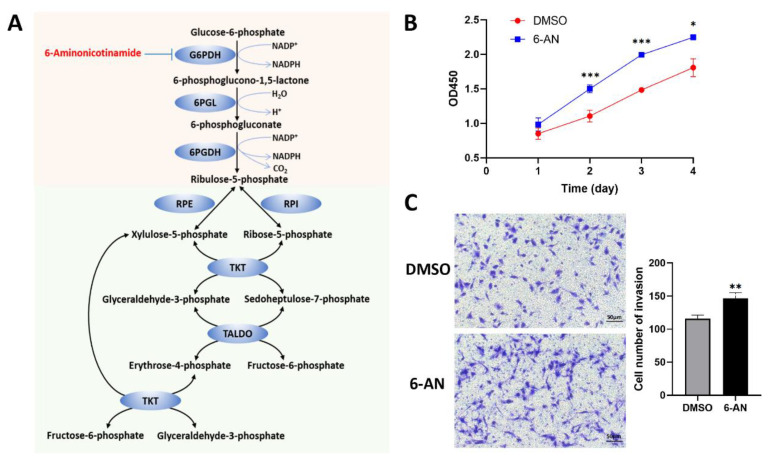
Ex vivo inhibition of PPP promotes cell proliferation and invasion of LPS853. (A) The pentose phosphate pathway and its inhibitor 6-AN. (**B**) The effects of 6-AN on the proliferation of LPS853 were determined via CCK-8 assays. (**C**) The effects of 6-AN on the invasion of LPS853 were determine via Transwell assays. Representative images of cell invasion and statistical analysis of specific invaded cell numbers were shown. *, *p* < 0.05; **, *p* < 0.01; ***, *p* < 0.001. G6PDH, glucose-6 phosphate dehydrogenase; 6PGL, 6-phosphogluconase; 6PGDH, 6-phosphogluconate dehydrogenase; RPE, ribulose-5-phoshate epimerase; RPI, ribulose-5-phoshate isomerase; TKT, transketolase; TALDO, transaldolase.

As the GSEA indicated that PPP was upregulated in WDLPS tumors, we then intended to examine if the PPP perturbation could alter the LPS tumor cell behavior. PPP is required for the synthesis of ribonucleotides and is a major source of NADPH (Figure 4A). It has been reported to be critical for cancer cells, since it generates pentose phosphates and NADPH required by the nucleic acid synthesis and cell survival of cancer cells[17]. To confirm whether the inhibition of PPP was involved in the development of LPS, we treated the ex vivo LPS cell line LPS853 with a PPP inhibitor, 6-aminonicotinamide (6-AN), to see if the cell behavior would turn more aggressive. Cell Counting Kit (CCK)-8 assays showed that 6-AN significantly promoted the proliferation of LPS853 cells (Figure 4B). Then, we investigated the potential of 6-AN in regulating LPS cell invasion. Transwell assays indicated that the invasion of LPS853 was promoted after treatment of 6-AN (Figure 4C). These results suggested that the inhibition of PPP on LPS cell led to a more aggressive tumor cell behavior. Such aggressive tumor cell behavior could be associated to the LPS cell dedifferentiation. Multiple researches reported that PPP played a role in the differentiation of adipocytes[18,19]. We therefore inferred that the perturbation of PPP may impact the development of LPS by interfering the adipocytic differentiation of LPS cell.

## 3. Discussion

LPSs are rare malignancies of adipocytic origin and abnormal metabolic activity may take place during their tumorigenesis. Increasing evidence has indicated that the tumorigenesis of WDLPS and DDLPS correlates with the abnormal adipogenic differentiation. However, the mechanisms underlying these correlations remain unclear, and there have been no effective adipogenesis-inducing drugs for LPS treatment. Previous studies have explored the role of differentiation therapy in treating LPS. Peroxisome proliferator activated receptors (PPARs) are critical transcription factors involved in adipocyte differentiation. PPARγ has been considered more related to the other LPS type, myxoid round cell liposarcoma (MRCLS). MRCLS features a translocation leading to the fusion protein TLS:CHOP, the tumorigenicity of which was reported to be a result of the suppression of PPARγ activity [20]. It was found that PPARγ agonists could enhance the adipose differentiation initiated by ET-743 in MRCLS [20]. PPARγ was found to be over-expressed in DDLPS by immunostaining too [21] and PPARδ was also found highly expressed in LPS compared to lipoma [22]. It has been shown that the agonists of PPARγ could induce human LPS cells to undergo terminal differentiation ex vivo [23]. While the stimulation of PPARδ was proved to promote the proliferation and migration of LPS cell lines, which could be inhibited by leptin, suggesting that the inhibition of PPARδ might be a potential strategy to treat LPS [22]. Another study found that the differentiation therapy consisting of four compounds, dexamethasone, isobutylmethylxanthine (IBMX), indomethacin, and insulin, could induce adipogenic differentiation of DDLPS cells and inhibit tumor growth both ex vivo and in vivo [24]. There were also clinical trials investigating the effect of PPARγ agonists on patients with LPS, but the antitumor activity was not significant in general [25,26].

The different adipogenic differentiation stages are always associated with distinct metabolic feature alterations, especially that of lipid metabolism. The interest on cancer metabolism has been increasing for other cancer types such as breast, liver, colorectal, and lung cancer [27,28,29], but is relatively sparse in sarcoma. Braas et al. performed metabolic footprint analysis on LPS cell lines, and revealed that nucleoside salvage activity was elevated in LPS, which may have association with the patients’ response to the nucleoside-based prodrug gemcitabine [30]. Another study by Patt et al. conducted metabolomic and lipidomic analysis on the patient-derived DDLPS cell lines [31]. A total of 17 metabolites, including ceramides, glycosylated ceramides, and sphingomyelins were found to be altered between cells with higher and lower *MDM2* amplification. They suggested that lipid metabolism may lead to the higher aggressiveness of upper *MDM2*-expressing DDLPS. These studies have contributed to revealing the metabolic features of LPS but they limited to the cell lines, instead of the LPS tissues, which has limitations in reflecting the true metabolic status of LPS tumors.

In our study, we performed the lipidomic analysis based on LC-MS/MS using the LPS tissues, and compared the lipid profiles of WDLPS and DDLPS for the first time. We observed from the lipidome that the WDLPS tumors consisted of great amount of neutral lipids such as TG and DG, which was similar to the composition of normal adipose tissue. This was in accordance with the histological appearance of WDLPS, which was mainly composed of cells resembling mature adipocytes. These observations suggested that WDLPS had a higher similarity to the mature adipose tissue. On the contrary, the lipid of DDLPS tumors consisted of more phospholipids, such as PC, PE and SM. Phospholipids, as the building-block components of cellular membranes, play key roles in the cell division, signaling and homeostasis of malignant cells [32]. It is inferred that the increased amount of phospholipids in DDLPS was associated to its higher amount of atypical cells, thus leading to its higher aggressiveness. The respective similarity of WDLPS and DDLPS to SVF cells of different time points further confirmed the hypothesis that the two subtypes were at the different differentiation stages.

According to the transcriptomic analysis, PPP was found to be upregulated in WDLPS. Theoretically, since the products of PPP including NADPH and ribose-5-phosphate play important roles in regulating DNA damage response, metabolism, and proliferation of cancer cells, PPP is usually considered critical for tumor survival and development. Various enzymes of the PPP have been reported to be elevated in many cancer types including lung, liver, colorectal and pancreatic cancer [33,34,35,36] and be identified as potential targets for cancer therapy. It is interesting that Na et al. recently found the depletion of PPP enzyme, transketolase (TKT) in mouse adipocyte could attenuate obesity happening [18]. In addition, Shantz et al. discovered the inhibition of glucose-6-phosphate dehydrogenases (G6PDs) could also block the differentiation of 3T3-L1 [19]. These two results indicated that the inhibition of PPP in LPS cell in our study did not function as an anticancer strategy, but an inhibition of adipocytic differentiation. As we showed in our lipidomic results, the main differences between two LPS tumors were their adipocytic differentiation stages, the perturbation of such differentiation stages could also manipulate the behavior of LPS tumor cell. We identified the different metabolic features between WDLPS and DDLPS for the first time and proposed that PPP may associate with the differentiation status of the two subtypes.

Honestly, there are some limitations in our current study which should be further addressed in the following research. First of all, we only included 13 samples in this study and a cohort with a larger sample size is required for validation in the future. We have not analyzed the correlation between the metabolic features and the clinical outcomes of the patients so the follow-up data should be completed and integrated to the current study. Second, the SVF cells previously reported by our group were isolated from mice and were used in this study since the crucial gene features of adipogenic differentiation were similar across *homo sapiens* and *mus musculus*. However, it is more appropriate to validate the current result using the transcriptome of human SVF cells in the future. Finally, although genes related to PPP were found upper-expressed in WDLPS tumors, the higher expression of key enzymes of PPP has not been observed in these samples. The PPP inhibitor 6-AN used here targeted the upstream enzyme G6PDH but the mechanism of its inhibition on LPS cell line was not elucidated in our study. In addition, the mechanism underlying its promoting function on the proliferation and invasion of LPS cell line, which was different from other cancer types, was not clear. Further experiments are needed to validate our hypothesis that the inhibition of PPP may promote a more malignant phenotype of LPS cell by blocking its adipose differentiation. In summary, a larger sample size and a better-established model is required for the future research. Deeper insights into the mechanism underlying the impact of PPP on LPS are necessary.

## 4. Materials and Methods

### 4.1. Sample Collection

All samples used in this study were derived from patients who underwent resection for retroperitoneal liposarcoma in Zhongshan Hospital, Fudan University (Shanghai, China). Patients over 18 years old and pathologically diagnosed with WDLPS or DDLPS were eligible for inclusion. Patients were firstly selected according to their preoperative imaging and only patients with pure WDLPS or DDLPS as shown by the CT (Figure S6) were enrolled. Then, tumor samples and the paired normal adipose tissues of these selected patients were collected during the surgeries. The specimens were sliced into several small parts once they were resected from the gross tumor. One part was stored in formalin, which was in preparation for paraffin imbedding and HE staining. The other adjacent two parts were frozen in liquid nitrogen in preparation for lipidomic or transcriptomic analysis respectively. The tumor was then pathologically confirmed as WDLPS or DDLPS by the Department of Pathology of Zhongshan Hospital. Then, the WDLPS samples that purely consisted of adipose-like cells and the DDLPS samples that purely consisted of spindle cells were selected according to the HE staining appearance. All patients were enrolled according to the tissue acquisition protocol approved by the Institutional Review Board of Zhongshan Hospital. Informed consent was obtained from all patients prior to sample collection. The baseline characteristics of the enrolled patients are detailed in Table 1.

### 4.2. Ex Vivo Cell Culture

The human LPS cell line LPS853 was a generous gift from Professor Yuexiang Wang of Shanghai Institute of Nutrition and Health, Chinese Academy of Sciences (Shanghai Branch, Shanghai, China) and Professor Jonathan A. Fletcher of the Department of Pathology, Brigham and Women’s Hospital, Harvard Medical School. The cells were cultured in IMDM medium (Gibco, USA) containing 10% fetal bovine serum (FBS) (Gibco, USA) and 1% penicillin/streptomycin (Gibco, USA), at 37 °C with 5% CO_2_.

### 4.3. Cell Proliferation Assay

A total of 1000 cells were seeded into each well in the 96-well plates and allowed to attach overnight. Cells were then treated with 1 μM 6-AN (MedChemExpress, USA). Dimethyl sulfoxide (DMSO) was used to dissolve 6-AN and the solution was diluted to the final concentration in IMDM supplemented with 10% FBS. Cells in the control group were treated with vehicle (DMSO in IMDM supplemented with 10% FBS). Cell proliferation was determined using the CCK-8 assay (Yeasen, Shanghai, China), according to the manufacturer’s instructions. A volume of 10 μL of CCK-8 was mixed with 90 μL of culture medium at 24, 48, 72, and 96 h after treatment and incubated at 37 °C for 2 h. Cell proliferation was measured using the microplate reader and the absorbance was measured at 450 nm.

### 4.4. Transwell Invasion Assay

To evaluate the invasion ability of LPS853 treated with 6-AN, Transwell assays were performed by using a 24-well Transwell plate (8 μm pore size; Corning, USA) coated with Matrigel (30 μg; BD Biosciences, USA). Tumor cells of 5 × 10^4^ in 200 μL of serum-free IMDM medium treated with 1μM 6-AN or DMSO were seeded in the upper chamber, and 600 μL of complete IMDM medium was added to the lower chamber. The plate was then incubated at 37 °C for 24 h. Subsequently, cells that invaded from the top to the bottom of the chamber were fixed in 4% paraformaldehyde for 30 min, stained with crystal violet solution (Beyotime, China) for 20 min, counted and photographed with a microscope in 5 independent 10× fields for each well. Three separate experiments were performed.

### 4.5. SVF Differentiation

The primary progenitor SVF cells were isolated and cultured as previously described[14]. Briefly, the subcutaneous adipose tissues were obtained from C57BL/6J mice and digested using collagenase D (10 mg/mL) (Sigma, St Louis, MI, USA) with bovine serum albumin (10 mg/mL) (Sigma, USA), incubated on a shaker at 37 °C, at 750 rpm, for 1 h. Then the samples were washed with DMEM medium and filtered through a 100 µm nylon mesh several times, and seeded in 24-well plates, cultured in a humidified CO_2_ incubator at 37 °C. 6 plates marking six time points every 2 days from 0th day to 10th day were prepared. To differentiate the primary SVF cells, a cocktail containing 5 mg/L insulin (Sigma, USA), 1 μM 3-isobutyl-1-methylxanthine (Sigma, USA), 0.5 nM dexamethasone (Thermo, Waltham, MA, USA) and 1 nM rosiglitazone (Sigma, USA) was added to the cell culture plate the 2nd day; 5 mg/L more insulin was added on the 4th day; and cells were kept in such media composition for the following 6 days. The RNA and lipids of SVF cells on each time points were collected using TRIzol and a methyl tert-butyl ether (MTBE)/methanol/water solution, respectively.

### 4.6. Lipid Extraction

Lipid extraction was performed following a published protocol [37]. Briefly, tumor samples were ground with 200 μL of water and 500 μL of methanol by a tissue-lyser at 4 °C. The homogenate was added to another 500 μL of methanol and decanted into a clean glass centrifuge tube. A total of 5 mL of MTBE was then added to the glass centrifuge tube and vortexed for 1 min. The glass centrifuge tube containing the homogenate was rocked on a shaker for 1 h at room temperature. A total of 1.25 mL of water was then added to the glass centrifuge tube followed by another minute of vortexing. The homogenate was centrifuged at 4 °C at 1000× *g* for 10 min and two-phase layers could be observed in the glass centrifuge tube. A total of 4 mL of the top phase supernatant were collected and dried under a stream of nitrogen. The extracted lipid samples were stored at −80 °C before LC-MS/MS analysis.

### 4.7. Lipidomic Analysis

Lipid samples were resuspended in 100 μL of chloroform:methanol:water (v:v:v, 45:45:10), and 10 μL was injected into a Orbitrap Exploris 480 LC-MS/MS (Thermo, USA) coupled to HPLC system (Shimadzu, Kyoto, Japan). Lipids were eluted via C30 by using a 3 m, 2.1 mm × 150 mm column (Waters) with a flow rate of 260 μL /min using buffer A (10 mM ammonium formate at a 60:40 ratio with acetonitrile:water) and buffer B (10 mM ammonium formate at a 90:10 ratio with isopropanol:acetonitrile). Gradients were held in 32% buffer B for 0.5 min and run from 32% buffer B to 45% buffer B at 0.5–4 min; from 45% buffer B to 52% buffer B at 4–5 min; from 52% buffer B to 58% buffer B at 5–8 min; from 58% buffer B to 66% buffer B at 8–11 min; from 66% buffer B to 70% buffer B at 11–14 min; from 70% buffer B to 75% buffer B at 14–18 min; from 75% buffer B to 97% buffer B at 18–21 min; 97% buffer B was held from 21–25 min; from 97% buffer B to 32% buffer B at 25–25.01 min; and 32% buffer B was held for 8 min. All the ions were acquired by non-targeted MRM transitions associated with their predicted retention time in a positive and negative mode switching fashion. ESI voltage was +5500 and −4500 V in positive or negative mode, respectively.

### 4.8. RNA Sequencing

Total RNA was extracted from tissues utilizing TRIzol reagent (Invitrogen, Waltham, MA, USA). The purity and concentration of RNA samples were determined using Nanodrop 2000 spectrophotometer (Thermo, USA). The integrity of RNA samples was evaluated using Agilent 2100 Bioanalyzer and 2100 RNA nano 6000 assay kit (Agilent Technologies, Germany). The RNA with poly-A in eukaryotic total RNA was enriched by TIANSeq mRNA Capture Kit (TIANGEN, Beijing, China) following the QC procedure. Then, the captured RNA was used to construct the transcriptome sequencing libraries utilizing TIANSeq Fast RNA Library Kit (Illumina, USA). Briefly, the construction of transcriptome sequencing library included random RNA fragmentation, cDNA strand 1/ strand 2 synthesis, end repair, A-tailing, ligation of sequencing adapters, size selection and library PCR enrichment. The library concentration was determined by the Qubit 2.0 fluorometer (Life Technologies, USA) and diluted to 1 ng/µL. Then, the insert size was checked on the Agilent 2100 and the library activity was quantified to greater accuracy (>2 nM) by quantitative PCR. The TruSeq PE Cluster Kit v3-cBot-HS (Illumina, USA) was utilized to perform the clustering of the index-coded samples on a cBot Cluster Generation System according to the manufacturer’s instructions. Then, the libraries were sequenced on an Illumina sequencing platform and 150 bp paired-end reads were generated. Raw data in the fastq format were firstly processed through in-house perl scripts. The clean data were obtained after removing the low-quality reads and those containing adapter and poly-N using Trimmomatic. Index of the reference genome was downloaded and paired-end clean reads were aligned to the reference genome using Hisat2. StringTie was applied to the bam file for expression quantification and the RNA-seq count data was obtained.

### 4.9. Metabolic Similarity between LPS and SVF

Batch effect between the transcriptomics of LPS and SVF was corrected using comBat function within the R packages sva [38]. Then, the distance between SVF (*x*) and LPS tumors (*T*) was calculated by the Euclidean distance ${\left|x-\bar{T}\right|}_{2}$ and normalized by the normal tissue (*N*). Next, the similarity between SVF cells and LPS tumors was calculate according to the equation:

Equation (1):

$$\mathrm{similarity}=\;\frac{1}{{\left|x-\bar{T}\right|}_{2}-{\left|x-\bar{N}\right|}_{2}}$$

### 4.10. Statistical Analysis

The lipidomic results were statistically analyzed with LINT-web[14]. Other statistical analysis was performed via R software V.4.0.2 (R Foundation for Statistical Computing, http://www.r-project.org/, accessed date: 10 October 2022) and GraphPad Prism 8.0 (GraphPad Software, San Diego, CA, USA). Two-tailed Student’s t-test, Kolmogorov−Smirnov test and Wilcoxon test was used for the indicated comparisons. *p* < 0.05 was considered statistically significant (* *p* < 0.05, ** *p* < 0.01, *** *p* < 0.001, ns, no significance).

## 5. Conclusions

Our study revealed the distinct lipid profiles of WDLPS and DDLPS by conducting lipidomic analysis based on LC-MS/MS. By comparing the transcriptome of LPS tumors and that of the SVF cells, we identified the different adipocyte differentiation stages of WDLPS and DDLPS. The transcriptomic analysis also suggested the upregulation of PPP in WDLPS tumors. The ex vivo inhibition of PPP using 6-AN promoted the proliferation and invasion of LPS cell line, which suggested that the inhibition of PPP may be one of the metabolic factors related to the different differentiation stages and biological behaviors between WDLPS and DDLPS.

## Figures and Tables

**Table 1:** Clinicopathological characteristics of liposarcoma patients.

Characteristics	WDLPS (N = 6)	DDLPS (N = 7)
	n (%)	n (%)
Age		
18–30	0 (0)	1 (14)
31–40	0 (0)	1 (14)
41–50	0 (0)	1 (14)
51–60	4 (67)	3 (43)
61–70	2 (33)	0 (0)
71–80	0 (0)	1 (14)
Gender		
Female	5 (83)	3 (43)
Male	1 (17)	4 (57)
Primary/Recurrent		
Primary	1 (17)	3 (43)
Recurrent	5 (83)	4 (57)
Previous surgery		
0	1 (17)	3 (43)
1	2 (33)	1 (14)
2	0 (0)	2 (29)
≥3	3 (50)	1 (14)
Other treatment prior to surgery		
None	5 (83)	3 (43)
Targeted therapy	1 (17)	1 (14)
Chemotherapy +Targeted therapy	0 (0)	1 (14)
Chemotherapy +Radiation therapy +Targeted therapy +Immunotherapy	0 (0)	2 (29)
FNCLCC grade		
I	6 (100)	0 (0)
II	0 (0)	4 (57)
III	0 (0)	3 (43)

## Data Availability

The names of the repositories and accession numbers can be found in the article/Supplementary Material.

## Supplementary Materials

The following supporting information can be downloaded at: https://www.mdpi.com/article/10.3390/metabo12121227/s1, Figure S1: Gross pictures and hematoxylin and eosin (H&E) staining of WDLPS and DDPS samples. (A) WDLPS tumors were mainly composed of mature adipocytes and intersected by fibrous septa and sparse spindle cells. (B) DDLPS tumors were mainly composed of high-grade spindle cells, with no apparent adipocytic differentiation. Scale bar=100μm.; Figure S2. Lipidomic analysis of WDLPS and lipoma. (A) PCA plot of the lipidome of WDLPS and lipoma compared with normal fat. (B) Venn diagram of the upregulated and downregulated lipids common to WDLPS and lipoma compared with normal fat; Figure S3. Lipid composition of WDLPS and lipoma. (A) Comparison of lipid composition between normal fat and WDLPS. (B) Comparison of lipid composition between normal fat and lipoma; Figure S4. Altered metabolism related pathways in WDLPS compared to DDLPS via GSEA; Figure S5. The expression level of some PPP related genes in WDLPS, DDLPS, normal fat and lipoma samples by RT-qPCR. (A) The expression level of these selected genes was higher in WDLPS than in DDLPS. (B-C) The expression level of these selected genes was similar among WDLPS, normal fat and lipoma. *, *p* < 0.05; **, *p* < 0.01; Figure S6. The representative imaging of WDLPS and DDLPS. (A) The representative imaging of pure WDLPS (red arrow). (B) The representative imaging of pure DDLPS (blue arrow). (C) The representative imaging of lesion with both WD (yellow arrow) and DD (green arrow) component; Table S1: Lipidomics Analysis of LPS; Table S2: Transcriptomics Analysis of LPS; Table S3: Transcriptomics Analysis of normal tiusse of LPS patients.
