# Supplementary material for: Lipidomics and Transcriptomics Differ Liposarcoma Differentiation Characteristics That Can Be Altered by Pentose Phosphate Pathway Intervention
Source: Metabolites. 2022 Dec 7;12(12):1227. doi: 10.3390/metabo12121227 (PMC9783184; doi:10.3390/metabo12121227)
Supplement: Supplementary file 1 [file metabolites-12-01227-s001.zip › Supplementary Figures.pdf]

Lipidomics and Transcriptomics Differ Liposarcoma Differentiation Characteristics that can be Altered by Pentose Phosphate Pathway Intervention

Zhengqing Song[1#], Shuaikang Wang[1#], Lili Lu[1#], Jingshen Xu[1], Qiwen Zhou[1], Weiqi Lu[1], Hanxing Tong[1], Yong Zhang[1], Wenshuai Liu[1], Zhiming Wang[1], Wei Li[1], Yang You[1], Chenlu Zhang[1], Xi Guo[1], Rongkui Luo[1], Yingyong Hou[1], Chunmeng Wang[2], Yuexiang Wang[3], Lei Sun[1]*, He Huang [1,4]*, Yuhong Zhou[1]*

[1] Department of Medical oncology, Biotherapy center, Department of General Surgery, Department of Pathology, Zhongshan Hospital, Shanghai Key Laboratory of Metabolic Remodeling and Health, Institute of Metabolism and Integrative Biology, Institute of Developmental Biology and Molecular Medicine, Fudan University, Shanghai 200032, China;

[2] Department of Musculoskeletal Oncology, Fudan University Shanghai Cancer Center, Shanghai 200032, China;

[3] Institute of Nutritional and Health Science, Chinese Academy of Sciences, Shanghai 200031, China;

[4] Shanghai Qi Zhi Institute, Shanghai 200030, China;

[#] These authors contribute equally;

* Correspondence: zhou.yuhong@zs-hospital.sh.cn (Y. Z.), he_huang@fudan.edu.cn (H. H.), lei_sun@fudan.edu.cn (L. S.).

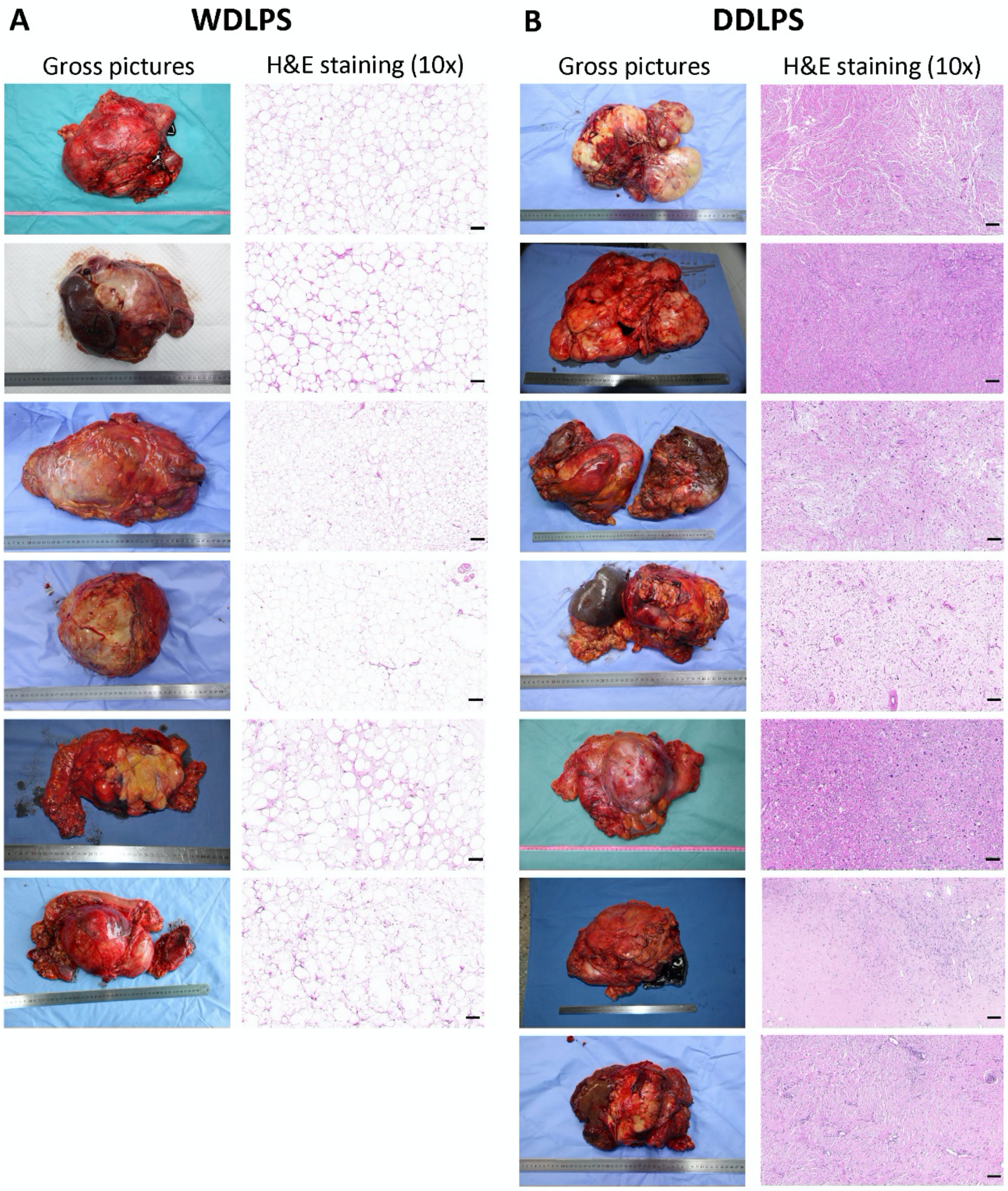

Figure S1. Gross pictures and hematoxylin and eosin (H&E) staining of WDLPS and DDPS samples. (A) WDLPS tumors were mainly composed of mature adipocytes and intersected by fibrous septa and sparse spindle cells. (B) DDLPS tumors were mainly composed of high-grade spindle cells, with no apparent adipocytic differentiation. Scale bar=100μm.

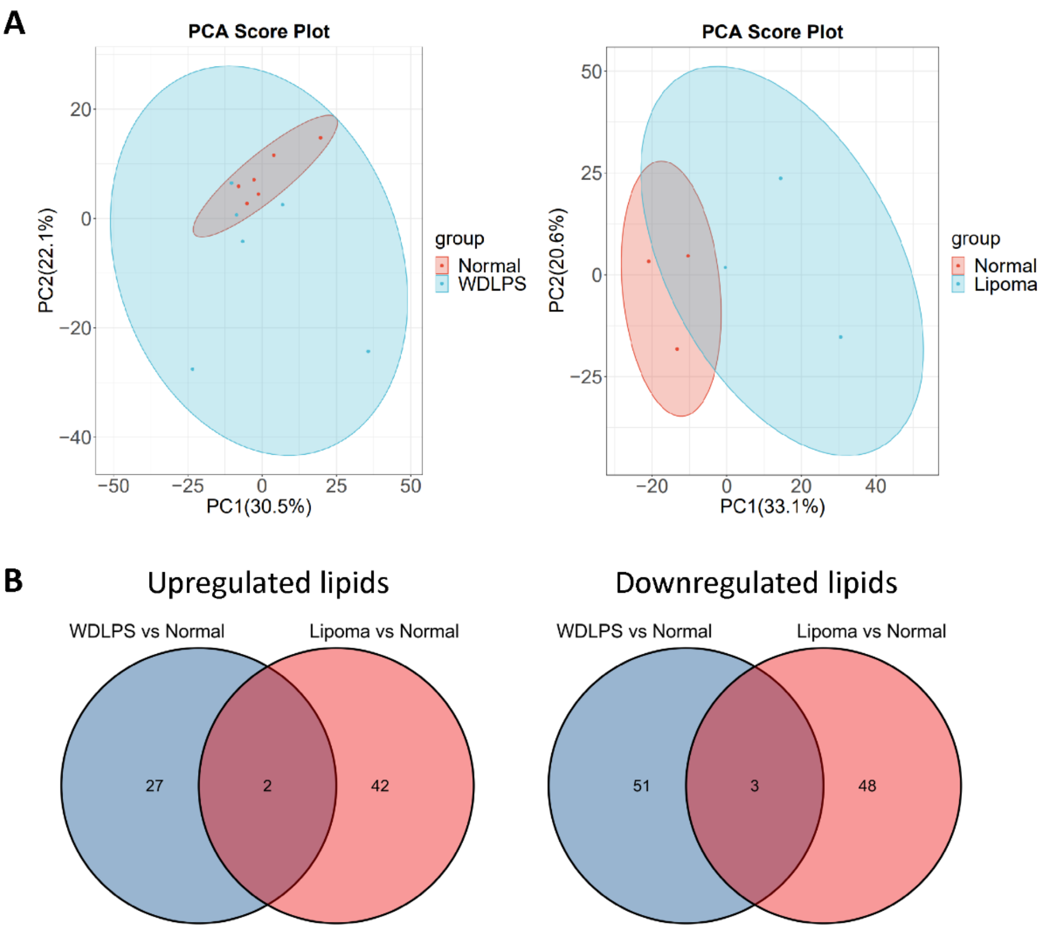

Figure S2. Lipidomic analysis of WDLPS and lipoma. (A) PCA plot of the lipidome of WDLPS and lipoma compared with normal fat. (B) Venn diagram of the upregulated and downregulated lipids common to WDLPS and lipoma compared with normal fat.

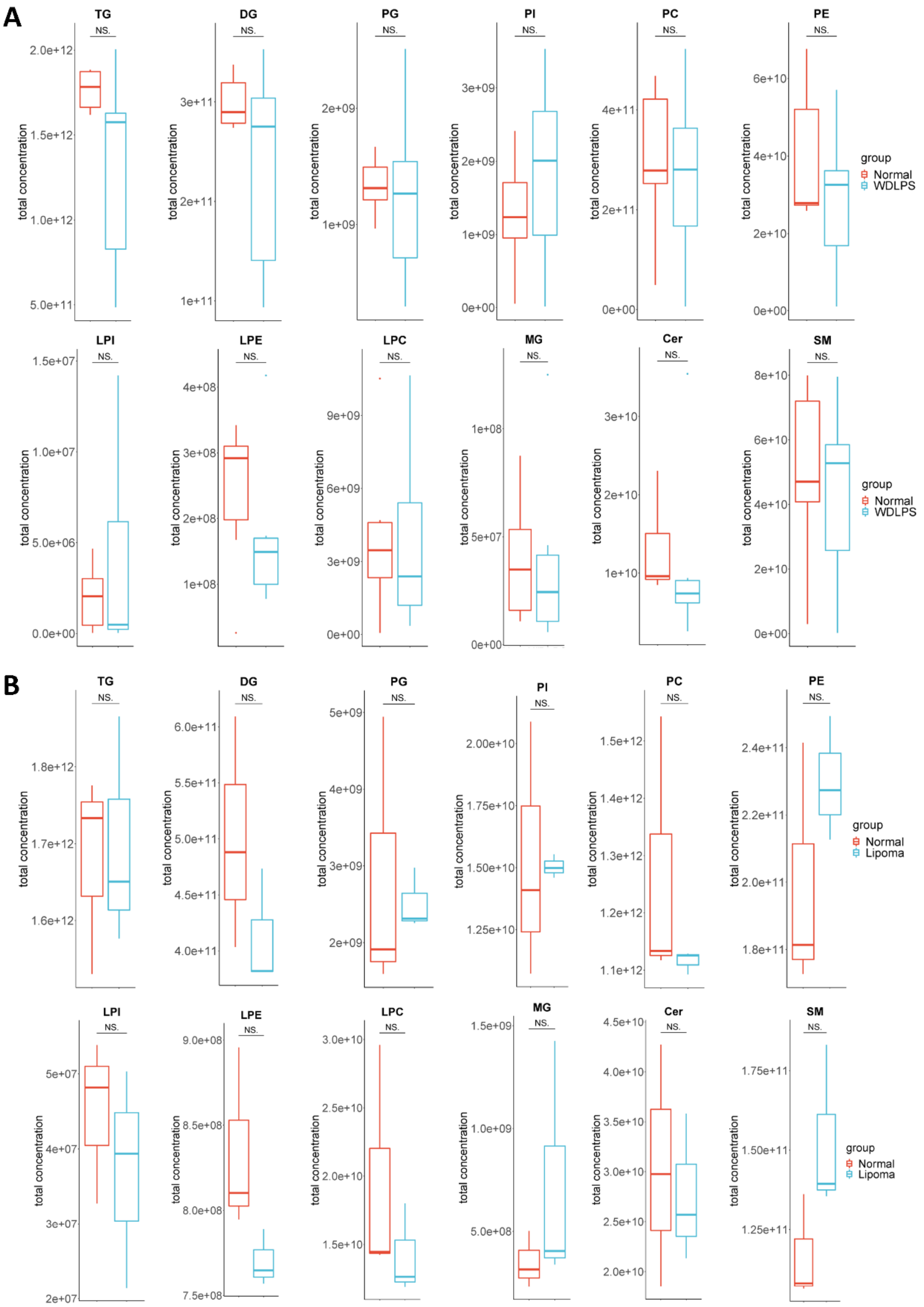

Figure S3. Lipid composition of WDLPS and lipoma. (A) Comparison of lipid composition between normal fat and WDLPS. (B) Comparison of lipid composition between normal fat and lipoma.

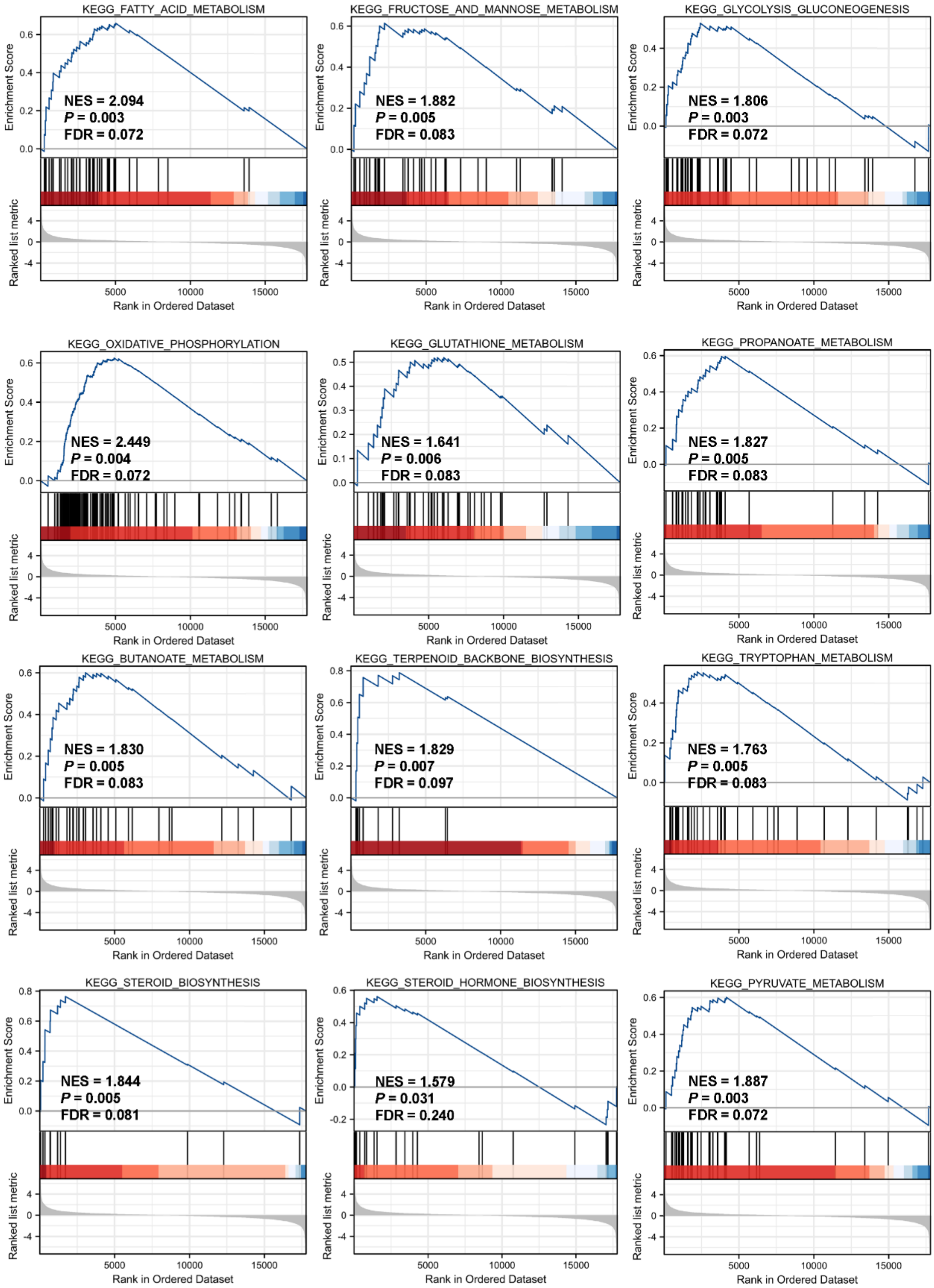

Figure S4. Altered metabolism related pathways in WDLPS compared to DDLPS via GSEA.

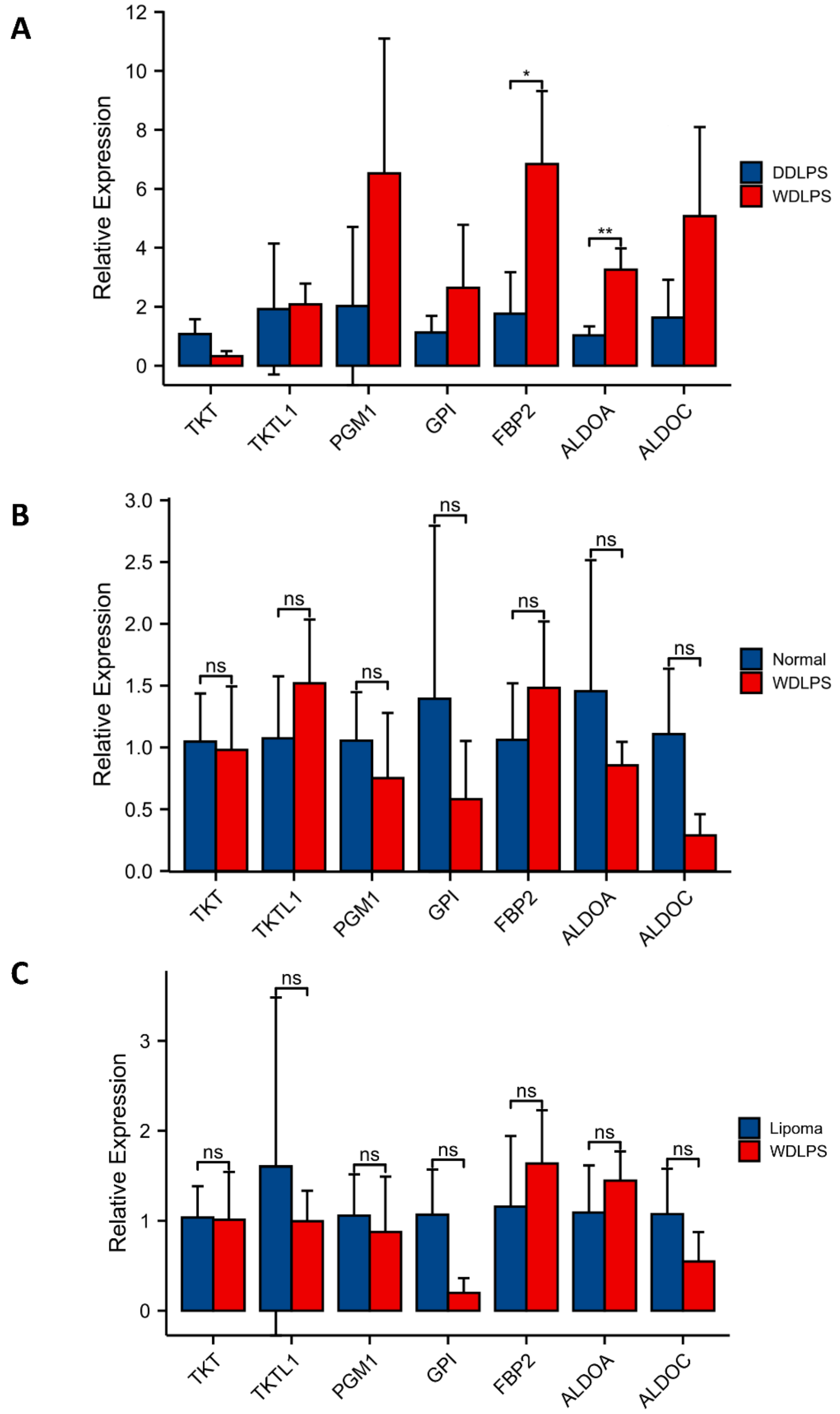

Figure S5. The expression level of some PPP related genes in WDLPS, DDLPS, normal fat and lipoma samples by RT-qPCR. (A) The expression level of these selected genes was higher in WDLPS than in DDLPS. (B-C) The expression level of these selected genes was similar among WDLPS, normal fat and lipoma. *, $P < 0.05$; **, $P < 0.01$.

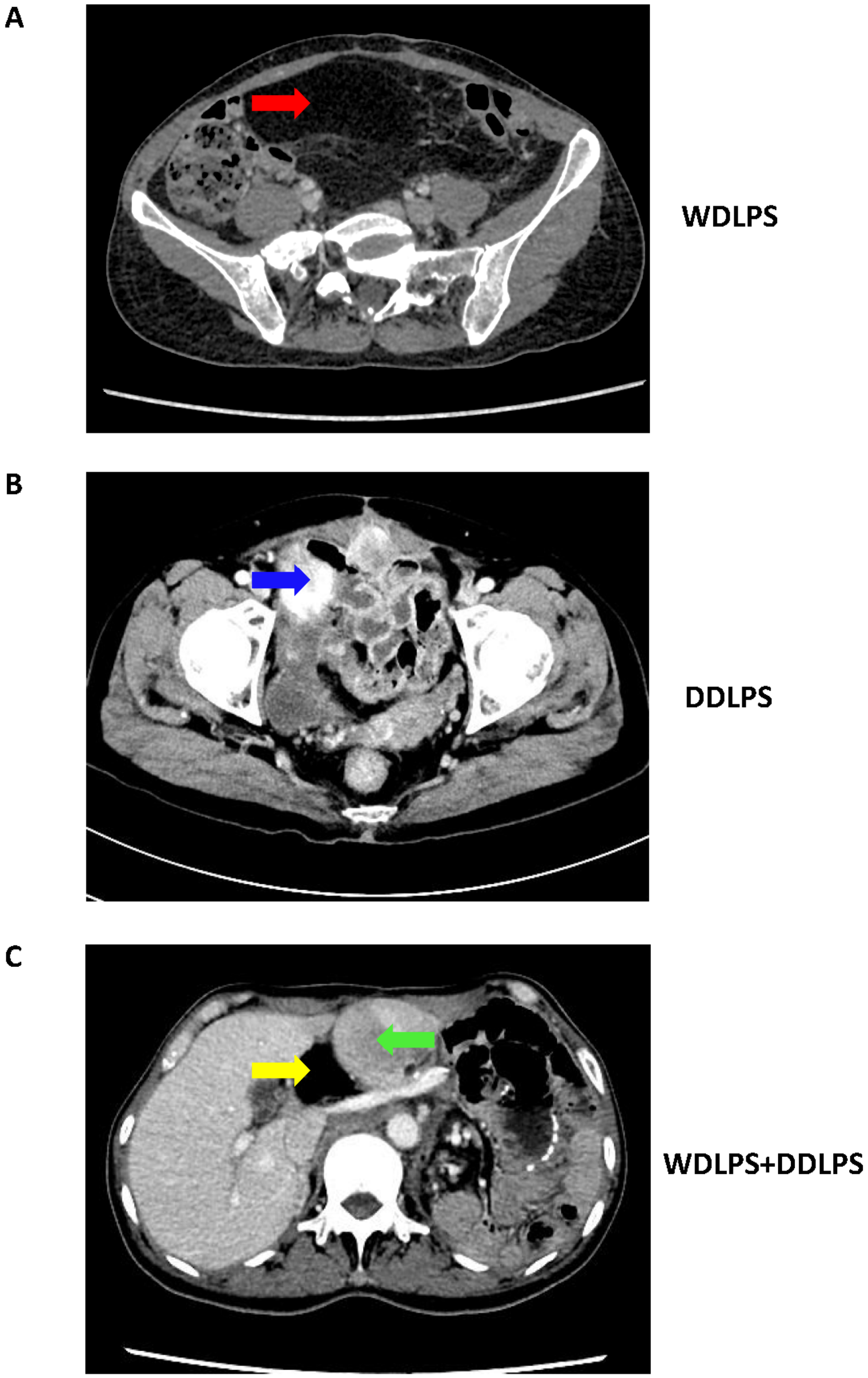

Figure S6. The representative imaging of WDLPS and DDLPS. (A) The representative imaging of pure WDLPS (red arrow). (B) The representative imaging of pure DDLPS (blue arrow). (C) The representative imaging of lesion with both WD (yellow arrow) and DD (green arrow) component.
